# Genome-wide association study, combined with bulk segregant analysis, identify plant receptors and defense related genes as candidate genes for downy mildew resistance in quinoa

**DOI:** 10.1186/s12870-024-05302-2

**Published:** 2024-06-24

**Authors:** Sara Fondevilla, Álvaro Calderón-González, Borja Rojas-Panadero, Verónica Cruz, Javier Matías

**Affiliations:** 1 Institute for Sustainable Agriculture, Consejo Superior de Investigaciones Científicas (CSIC), Avd. Menéndez Pidal s/n, Córdoba, 14004 Spain; 2 Agrarian Research Institute “La Orden-Valdesequera” of Extremadura, Centro de Investigaciones Científicas y Tecnológicas de Extremadura (CICYTEX), Autovía A-5, km 372 - 06187, Guadajira, Badajoz, 06187 Spain

**Keywords:** GWAS, Genetic resistance, *Peronospora variabilis*, *Chenopodium quinoa*

## Abstract

**Background:**

Downy mildew is the most relevant disease of quinoa and the most widespread. Though, little is known about the genetics of resistance to this disease. The objective of this study was to identify the genomic regions controlling downy mildew resistance in quinoa and candidate genes for this trait. With this aim we carried out a GWAS analysis in a collection formed by 211 quinoa accessions from different origins. This approach was combined with inheritance studies and Bulk Segregant Analysis (BSA) in a segregating population.

**Results:**

GWAS analysis identified 26 genomic regions associated with the trait. Inheritance studies in a F_2_ population segregating for resistance revealed the existence of a major single dominant gene controlling downy mildew complete resistance in quinoa accession PI614911. Through BSA, this gene was found to be located in chromosome 4, in a region also identified by GWAS. Furthermore, several plant receptors and resistance genes were found to be located into the genomic regions identified by GWAS and are postulated as candidate genes for resistance.

**Conclusions:**

Until now, little was known about the genetic control of downy mildew resistance in quinoa. A previous inheritance study suggested that resistance to this disease was a quantitative polygenic trait and previous GWAS analyses were unable to identify accurate markers for this disease. In our study we demonstrate the existence of, at least, one major gene conferring resistance to this disease, identify the genomic regions involved in the trait and provide plausible candidate genes involved in defense. Therefore, this study significantly increases our knowledge about the genetics of downy mildew resistance and provides relevant information for breeding for this important trait.

**Supplementary Information:**

The online version contains supplementary material available at 10.1186/s12870-024-05302-2.

## Background

Quinoa (*Chenopodium quinoa* Willd.) is a member of the Amaranthaceae family. Quinoa is a predominantly autogamous (self-pollinated) species with varying rates of natural hybridization (10–17%) [1]. It is an allotetraploid (2n = 4x = 36) but shows disomic inheritance for most qualitative traits [2]. Quinoa was initially domesticated by the indigenous civilizations of Bolivian and Peruvian Altiplano [3] and, subsequently, the crop has expanded to western South America. Two germplasm pools have been reported in quinoa: Andean highland quinoa, which is the primary center of diversity, and central and southern Chilean quinoa, the second center of diversity [4]. Through a process of selection and diversification, the species is now divided in five major ecotypes: Altiplano, Salar, Yunga, Valley and Lowland [5].

The exceptional nutritional characteristics of quinoa, coupled with its intrinsic tolerance to drought, salinity and frost has attracted worldwide attention to quinoa cultivation [6]. Quinoa provides all the essential amino acids required for humans [7–12], being also rich in minerals, vitamins, dietary fiber, linolenate, and natural antioxidants. For all these nutritional qualities quinoa is considered a “superfood” and its consumption has increased in the last years.

Quinoa remains an important food crop in South America, but, these desirable characteristics of quinoa have led to its cultivation expanding to numerous countries, being currently grown in more than 95 countries [5]. In Spain, quinoa cultivation started around 10 years ago, being now an emerging crop with about 6000 ha planted.

For a sustainable cultivation of quinoa, controlling quinoa diseases through an environment-friendly method, as genetic resistance, is desirable. This is especially relevant in this crop because consumers demand mainly organic quinoa. The main disease affecting quinoa worldwide is downy mildew, caused by the biotrophic oomycete *Peronospora variabilis* Gäum. Therefore, resistance to this pathogen is a key breeding target. *P. variabilis* infects the leaves of the plant. The initial symptoms are small, isolated chlorotic spots on the upper face of the leaves that later grow into irregular chlorotic spots, that finally become necrotic. On the underside of the leaves, the sporulation of the pathogen in the lesions produces the appearance of a greyish or purplish layer. In cases of severe infection, defoliation occurs. This disease can cause up to 99% yield losses in susceptible cultivars [13]. In Spain, this disease affects severely this crop, affecting up to 90% of the plant area and causing defoliation in susceptible cultivars under conditions especially favourable for the disease.

Downy mildew resistance in quinoa ranges from complete resistance to high susceptibility [13–16]. These observations suggest that resistance to *P. variabilis* on quinoa could be controlled by both major and minor genes, depending on the accession, but little is known about the inheritance of the trait. Benlhabib et al. [17] evaluated several traits, including, resistance to downy mildew, in a F_2:6_ quinoa population derived from a cross between the slightly susceptible accession NL-6 and the resistant accession 0654. Their results suggest that resistance in these lines is a polygenic trait, as around 50% of F_2:6_ families were classified between the two parents and transgressive segregation for resistance was observed, indicating that resistance could be controlled by different genes in the parental lines. The genetics of downy mildew resistance in quinoa has also been analysed in germplasm collections in two studies using Genome-Wide Association Studies (GWAS) [18, 19]. However, these studies were unable to identify markers associated with the trait, or the markers identified were not consistent. Therefore, further studies are needed to unravel the genetic structure of downy mildew resistance in quinoa and to identify the genes controlling the trait. The identification of these genes, and molecular markers linked to them, would facilitate their introduction and combination into susceptible varieties.

The aim of the present study was to unravel the genetics of resistance to this important disease in quinoa and identify candidate genes for the trait. With this aim, in this study we combined a GWAS, carried out in a germplasm collection formed by 211 quinoa accessions, with inheritance studies and a Bulk Segregant Analysis performed using a cross segregating for resistance. GWAS identified several genomic regions associated with downy mildew resistance in quinoa and a set of plant receptors and defense related genes, located into these regions, are postulated as candidate genes for this trait. Furthermore, resistance in accession PI614911 was found to be controlled by a single dominant gene that is located in chromosome 4, into a region also identified by GWAS.

## Methods

### Evaluation of the response to downy mildew in a quinoa germplasm collection

The response to downy mildew was scored under field conditions in a collection formed by 211 quinoa accessions with different geographical origins and covering the two main germplasm pools described in quinoa. Cutivars F16 and Kancolla were also included in the assays. The code and origin of these accessions is included in Supplementary Table S1. Seeds were obtained from the USDA North Central Regional Plant Introduction Station of the US National Plant Germplasm SystemUSDA (EEUU) and IPK Gatersleben (Germany) genebanks, excepting cultivar F16, that was provided by Algosur S.A. company. To ensure homogeneity of accessions, before performing the sequencing and field experiments, one plant from each accession was selected and selfed at least twice. An initial set of 138 accessions were screened in 2019 at experimental plots located in Córdoba (37°53′4.226″N 4°46′46.443″W) (Spain) and the whole collection was screened during 2021 and 2022 in Córdoba and Guadajira (38°51’07’’ N, 6°40’49’’ O) (Spain). Accessions were sown according to a completely randomised block design with three blocks. In each block each accession was represented by a 1 m row (10 plants per row) separated 0.7 m from the other rows. Basal fertilization (400 kg/ha of 8:15:15 N: P:K fertilizer plus 87 Kg of urea/ha) was applied before sowing and top dressing (130 kg urea/ha) at flowering.

The severity of the disease, estimated as the percentage of the plant’s leaf area with symptoms, was scored using the “three-leaf screening method”, which considers the average percentage of leaf area in each plant that is infected by the pathogen in three leaves randomly selected: one from each of the lower, middle and upper part of the plant [20]. Disease severity was evaluated once a week, from the time the first symptoms appeared until the senescence of the plant made it difficult to distinguish the symptoms caused by downy mildew from those caused by senescence. Disease severity in the last assessment was considered as final disease severity and used in the analyses.

The correlation between the severities obtained in the different environments was calculated using Pearson’s correlation coefficient.

### Genome sequencing and identification of genomic variations

For each quinoa accession forming the collection, DNA was isolated from frozen young leaf tissue obtained from plants grown in a greenhouse using “NucleoSpin Plant II” (Macherey-Nagel GmbH, Germany) kit. After checking its purity and quality by agarose gel electrophoresis, DNA concentration was determined using a Qubit instrument and optimum DNA samples were sent to Diversity Array Technology Pty Ltd (Camberra, Australia) for sequencing and genotyping as described in [21]. Briefly, DNA samples were processed as follows: PstI and MseI compatible adaptors with two different restriction enzyme overhangs were added [22]. The PstI and MseI compatible adaptors were designed to include the Illumina flowcell attachment sequence, sequencing primer sequence and “staggered”, varying length barcode region, similar to the sequence reported by [23]. The reverse adaptor contained the flowcell attachment region and MseI compatible overhang sequence. Only “mixed fragments” were effectively amplified in 30 rounds of PCR using the following reaction conditions: 94 °C for 1 min; 30 cycles of: 94 °C for 20 s, 58 °C for 30 s, 72 °C for 45 s; 72 °C for 7 min. After PCR, equimolar amounts of amplified product were bulked and subjected to 100 cycles of sequencing (single reads) on the Illumina Illumina NovaSeq sequencer. Sequences generated from each lane were processed using proprietary DArT analytical pipelines. In the initial pipeline, poor quality sequences were removed, with more stringent filtering parameters applied to the barcode region compared to the rest of the sequence, ensuring the assignments of the sequences to specific samples (based on the “barcode split”) was reliable. Filtering was performed on the raw sequences using the following parameters: Barcode region minimum Phred score 30, minimum percentage 75; whole read minimun Phred score 10, minimum percentage 50. Approximately 340,412 unique sequences per sample were used in marker calling. Identical sequences were collapsed into “fastqcoll files” which were “groomed” using DArT PL’s proprietary algorithm which corrects low quality base from singleton tag into a correct base using collapsed tags with multiple members as a template. The “groomed” fastqcol files were used in the secondary pipeline for DArT PL’s proprietary SNP and SilicoDArT (presence/absence of restriction fragments in representation) calling algorithms (DArTsoft14). For SNP calling, all tags from all libraries included in the DArTsoft14 analysis are clustered using DArT PL’s C + + algorithm at the threshold distance of 3, followed by parsing of the clusters into separate SNP loci using a range of parameters, including the balance of read counts for the allelic pairs. Additional selection criteria were added to the algorithm based on analysis of approximately 1,000 controlled cross populations. Testing for Mendelian distribution of alleles in these populations facilitated selection of technical parameters discriminating true allelic variants from paralogous sequences. In addition, multiple samples were processed from DNA to allelic calls as technical replicates and scoring consistency was used as the main selection criteria for high quality/low error rate markers. Markers identified (SNPs and SilicoDArT) were assigned to chromosomes using version one of quinoa reference genome (CoGe id33827).

### Population structure analysis

The software fastSTRUCTURE [24] was used to estimate the number of populations (K) represented in the data. The input used was a reduced set of SNPs with linkage disequilibrium r² < 0.2 computed on a window of fifty markers that shifts five at the end of each step. PLINK 1.9 [25] was used for this filtering.

In addition, population structure was also inferred by Principal Component Analysis using GAPIT 3.1.0.

To test whether there were significant differences in ‘final disease severity’ between the different populations predicted by PCA and fastSTRUCTURE two analyses of variance (one for each software) were performed. In addition, in the case of the populations predicted by fastSTRUCTURE, as there were more than two populations, comparisons of mean values were performed by least significant difference (LSD) test. These analyses were carried out using the Statistix 8.0 package (Analytical Software, Tallahassee, FL, USA).

### Linkage disequilibrium (LD) analysis

LD and squared correlation coefficients (r²) between SNPs within a sliding window of fifty SNPs were computed using TASSEL 5.0 [26]. LD decay and LD half decay distance were estimated using Hill and Weir [27, 28] formula in R [29].

### GWAS analysis

First, in order to obtain accurate results, markers that could not be assigned to any chromosome and those that showed more than 20% missing values were excluded. GWAS analyses were carried out by GAPIT 3.1.0 and TASSEL 5.0 software. To take population structure and kinship into account, TASSEL 5.0. was used to obtain a Q matrix (using the multidimensional scaling (MDS) method) and a kinship matrix, that were subsequently used in GAPIT. Several models: MLM (Mixed Linear Model), GLM (General Linear Model), MLMM (Multiple Loci Mixed Model), FarmCPU and BLINK were used. MLM model was analysed using Tassel software while the rest of models were analysed using GAPIT software. “Model Selection” tool, as implemented by GAPIT, was used to determine the optimal number of PCs (covariates) to include for each phenotype. Multiple testing was corrected using Benjamini and Hochberg [30] false discovery rate (FDR) [31] (q = 0.1). Furthermore, quantile-quantile plots (QQplots) were obtained for each model and only when the QQplots showed that the data fit the model, the resulting marker-trait associations (MTAs) were considered accurate. In QQplots, the observed - log10 (P) for each marker are plotted against expected - log_10_ (P) values under the null hypothesis (no association of the markers with the trait). It is expected that only a few markers would be associated with the trait that is being evaluated. Therefore, if a model is suitable for analysing the data, in the QQplot most of the markers should be on or near the middle line between the x-axis and the y-axis and only a few (those associated with the trait) will be far from this middle line. In addition, MTAs were only considered reliable when their allele frequency was > 5%.

An analysis of variance was performed to check the effect of the factors ‘accession’, ‘location’ and ‘year’ on the variable ‘disease severity’. This analysis was performed using the software IBM SPSS statistic (version 26). As this analysis indicated that all factors, and their interactions, were significant (Suppl. Table S2), “disease severity” scored in each combination ‘location * year’ was considered a different trait and analysed separately in the GWAS analyses.

### Identification of candidate genes controlling resistance to downy mildew

To identify candidate genes controlling resistance to downy mildew in the quinoa collection, we searched in the version one of quinoa reference genome of cultivar QQ74 (NCBI code ASM168347v1) in a range of 250 kb down and upstream the MTAs identified by GWAS, using the browse tool available at GeGo website (https://genomevolution.org/CoGe/SearchResults.pl?s=quinoa&p=genome; CoGe id33827). This threshold was selected because it was the maximum range for LD half decay distance calculated across the different chromosomes in our study.

### Inheritance studies

The inheritance of resistance to *P. variabilis* was studied in an F_2_ population derived from the cross between the resistance accession PI614911 and the susceptible breeding accession Q122. The cross was made according to the method described by [32]. Q122 was used a female parent and PI614911 as male parent. In order to confirm that the seeds obtained were real F_1_, and not the result of self-pollination, a set of RAPD (Random Amplified Polymorphic DNA) markers were surveyed in the parental lines. DNA extraction and RAPD analyses were performed as reported in [33]. A primer OPC16 (Operon Technologies, Alameda California), showing polymorphism between the parents was next tested individually on DNA from the different F_1_ plants obtained. One F_1_ plant, showing bands from both parents, being, therefore the result of a real cross, was selfed in a greenhouse to obtain the F_2_ population. F_2_ plants were sown in the field and selfed to obtain the F_3_ families.

Resistance to *P. variabilis* in the Q122 x PI614911 F_2_ population and parental lines was evaluated in 2019 in an experimental plot located in Córdoba (Spain). Parental lines were sown in three replicates, having each 1 m row of each parent with 10 plants per row. To evaluate the F_2_ population, two hundred F_2_ seeds were sown in a row. F_3_ families were sown in rows having each ninety seeds of the corresponding family and evaluated during 2020 season. Plants were evaluated several times as described above and classified as resistant or susceptible according to their disease severity.

Goodness of fit to expected segregations was checked using chi-square tests.

### Bulk segregant analysis

According to the results obtained in the evaluation of downy mildew resistance in the F_2_ and F_3_ generations derived from cross Q122 x PI614911, seven F_2_ plants homozygous for resistance and ten plants homozygous for susceptibility were selected. Their DNA was extracted, as described above for the GWAS panel, and two pools, one formed by the resistance plants an another formed by the susceptible plants, were created mixing equal amount of DNA from each of the plants forming the pool. These pooled DNA samples were sent to Diversity Array Technology Pty Ltd (Camberra, Australia) for sequencing and SNP calling, as described above for the GWAS collection, but, in this case, each sample was sequenced twice. Those SNPs that could not be assigned to chromosomes were omitted and read depth information was used for BSA analysis using BSAvis software (https://github.com/FadyMohareb/BSAvis_GP_2020/tree/main/BSAvis). Briefly, a SNP index was calculated across the different chromosomes [34], those SNPs having a SNP index in both pools < 0.3 or equal to 1 were excluded, and an average SNP index was calculated using the sliding window method (window size of 1 Mb and a step size of 10 kb). ΔSNP index was further calculated as the difference between the SNP index of the two pools and a ΔSNP-index graphs was generated by plotting ΔSNP-index against the position in each chromosome.

The hypothesis is that, for a marker unlinked to the resistance gene, having 50% mutant and 50% wild-type sequence reads is expected, while the causal SNP, and closely linked SNPs, should show 100% mutant and 0% wild-type reads. SNPs loosely linked to the causal mutation should have > 50% mutant and < 50% wild-type reads. If we define the SNP index as the ratio between the number of reads of a mutant SNP and the total number of reads corresponding to the SNP, we expect that this index would be equal 1 near the causal gene and 0.5 for the unlinked loci [35] We further calculated the difference between the SNP index of the two pools to obtain the ΔSNP index. Δ SNP-index would be equal to 1 when the genome of bulked DNA is consistent with that of one of the parents, while Δ SNP-index will be − 1 when the genome of bulked DNA is consistent with that of the other parent, and Δ SNP-index = 0 when both parents had the same SNP-indices at the genomic regions. Thus, the Δ SNP-index value should be different from 0 if a genomic region harbours a target gene.

## Results

### Genotyping

The collection formed by 211 quinoa accessions was sequenced and genotyped using DArTseq technology. DArTseq is a genome complexity reduction-based sequencing technology (https://www.diversityarrays.com/services/dartseq/) that produces two types of markers: SNPs (Single Nucleotide Polymorphism) and SilicoDArT (presence/absence of the tags sequences). An average of 2,564,206 reads were obtained per DNA sample and used for marker calling. Marker calling quality was validated by high average read depth per locus (average across all markers was over 15.7 reads/locus). After eliminating those markers with low quality, those that could not be assigned to chromosomes and those having more than 20% missing values, a total of 12,397 SNPs and 12,720 SilicoDArT markers were selected and used in further analyses. The distribution of markers and the average distance between markers, per chromosome, is shown in Suppl. Table S3.

### Linkage disequilibrium analysis

LD half decay distance was estimated for each chromosome. This parameter varied between chromosomes ranging between 67,209 bp (in chromosome 13) and 248,075 bp (in chromosome 6) and showed an average value of 126,448 bp (Suppl. Fig. S1).

### Population structure

**Fig. 1:**
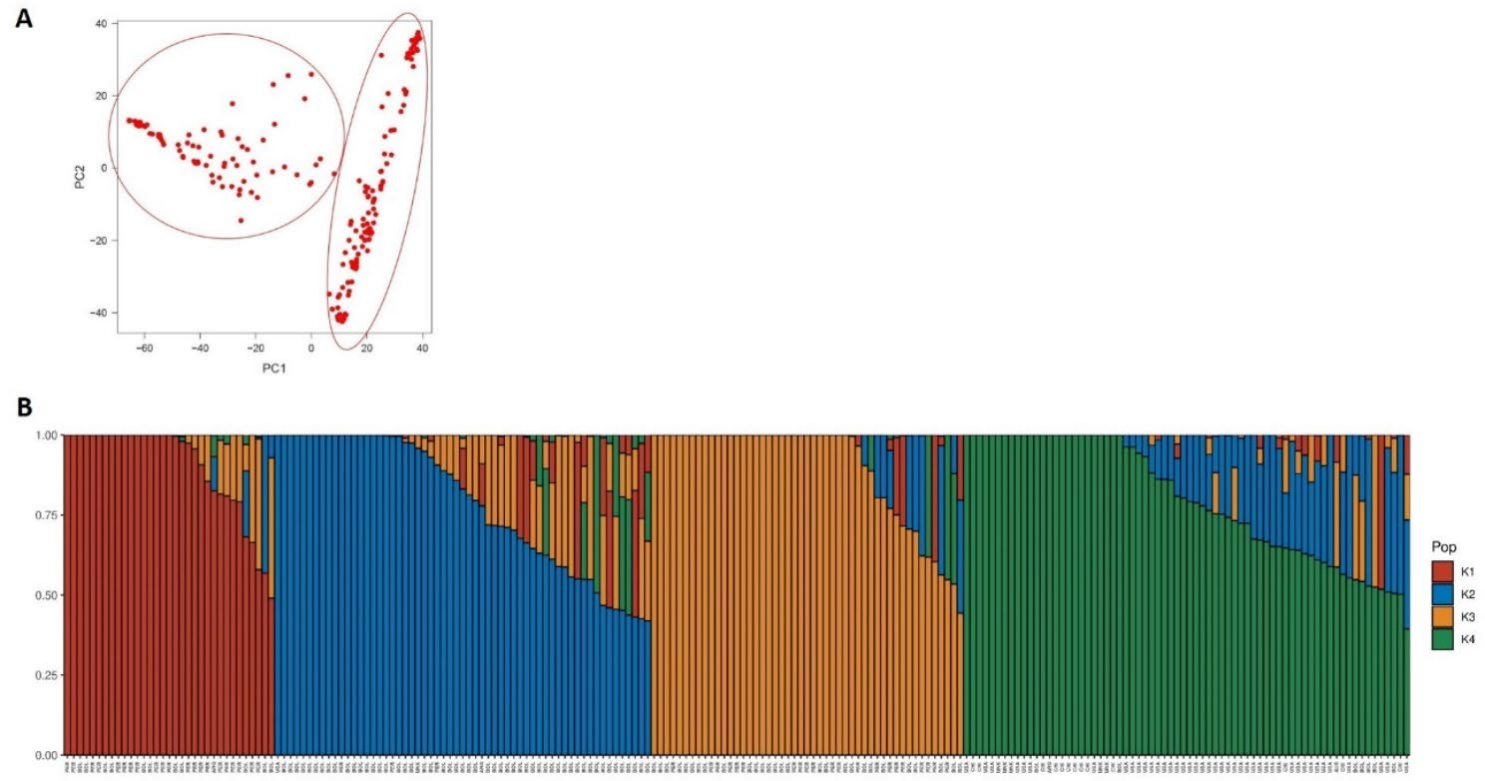
Population structure of the quinoa germplasm collection according to Principal Component Analysis. (**A**) and according to fastSTRUCTURE software (**B**)

Population structure in the quinoa collection was examined using Principal Components Analysis (PCA) and fastSTRUCTURE software. PCA, using the first and second principal components (that explained 25 and 11% of the variation, respectively) divided the collection into two main groups, in agreement with the two main germplasm pools reported for quinoa (Fig. 1A). Thus, one group was formed mainly by highland quinoa accessions (accessions mainly from Peru and Bolivia) and the other formed mainly by lowland quinoas accessions (accessions mainly from Chile and USA). Accessions from USA had been previously reported to be closely related with accessions from Chile as the USDA germplasm had been collected at these geographical regions [19].

fastSTRUCTURE software divided these two groups in additional subpopulations. So that, according to fastSTRUCTURE and the “chooseK.py” script included in it, that provides the value of K that best fits the data, the collection could be divided in four populations (Fig. 1B). One of this population corresponded to the PCA group containing the lowland accessions, while highland accessions were subdivided in three groups, one formed mainly by accessions from Bolivia, other formed mainly by accessions from Peru and a third one containing accessions from both Bolivia and Peru. These results show, in agreement with previous studies [19], that highland quinoa accessions show a wider genetic diversity than lowland accessions.

The assignment of the accessions included in the GWAS panel to each predicted population group can be seen in Suppl. Table S1.

### Response to downy mildew in a quinoa germplasm collection

**Fig. 2:**
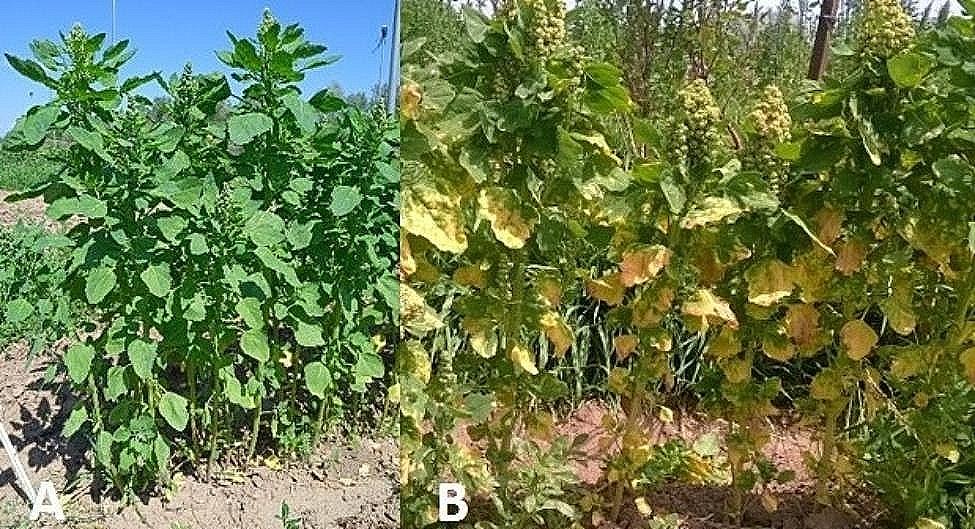
Quinoa accessions resistant to downy mildew (**A**) and susceptible to downy mildew (**B**)

Response to downy mildew was scored in a germplasm collection formed by 211 quinoa accessions during 2019, 2021 and 2022 in Córdoba (South of Spain) and during 2021 and 2022 in Guadajira (Central West Spain). The collection showed substantial phenotypic variation for this trait (Fig. 2). In all seasons disease severity showed a continuous distribution ranging from high resistance to high susceptibility, although disease was more severe in Córdoba than in Guadajira (Suppl. Fig. S2). So that, in Córdoba disease severity ranged from 0 to 82.5% of the plant area affected by the disease in 2019, from 2 to 73.3% in 2021 and from 0 to 73.3% in 2022. In Guadajira, disease severity ranged from 10 to 55% in 2021 and from 5 to 31.6% in 2022. These data show that downy mildew can severely affect the quinoa crop in Spain when the accession is susceptible and the conditions conductive for the development of the disease, but that, fortunately, genetic resistance to the *P. variabilis* isolates present in Spain is available within quinoa germplasm. Disease severity values were not correlated between locations but showed a significant correlation between the scorings carried out in the same location (Table 1).

**Table 1:** Pearson’s correlation coefficient between downy mildew disease severity values scored in a collection of 211 quinoa accessions in different environments

	Córdoba 2021	Guadajira 2021	Córdoba 2022	Guadajira 2022
**Guadajira 2021**	0.0023			
**Córdoba 2022**	0.3990 **	0.0120		
**Guadajira 2022**	-0.1678	0.5051**	-0.1999	
**Córdoba 2019**	0.3700 **	0.0199	0.8513 **	-0.1587

** the correlation is significant at *p* < 0.01

### GWAS and candidate genes analyses

GWAS analysis was performed using MLM, GLM, MLMM, FarmCPU and BLINK models. Resulting QQplots indicated that, in general, MLMM, FarmCPU and BLINK were a better fit to the data than MLM and GLM (Suppl. Fig. S3). A total of 58 MTAs, corresponding to 26 genomic regions, passes the quality criteria (good fit to the model, according to the QQplot, p-adjusted < 0.1 and MAF > 0.05) and, were, therefore, considered to be associated with resistance to downy mildew accurately in the quinoa germplasm collection. Several of these regions were identified in more than one environment, while others were specific for a certain environment. These regions are summarized in Table 2 and the exact location of each MTA can be found in Supplementary Table S4.

We then searched for candidate genes with a putative function in defense within 250 kb down and upstream the MTAs identified. Interestingly, most of the genomic regions surrounding the MTAs contained plant receptor or defense related genes, such as “receptor like proteins”, “disease resistance proteins”, “Wall-associated receptor kinases”, “pathogenesis like protein”, “Zinc finger BED domain-containing protein” and “L-type lectin-domain containing receptor kinase”, among others (Table 2; Suppl. Table S4). Remarkably, for ten of the MTAs, these candidate genes with a putative function in defense were located exactly in the same genomic position as the MTA (not in the surroundings 250 kb) (Suppl. Table S4).

**Table 2:** Genomic regions associated with downy mildew resistance in a quinoa germplasm collection identified by GWAS analysis. For each region, position on quinoa genome, the environment where it was identified, and candidate genes located into the region are shown

Chr	Position Mb	Environments	Candidate genes in the genomic region
1	1.16	G2021	Similar to HIN1 resistance gene
1	34.50-40.33	G2021	Serine/threonine-protein kinase; Receptor like protein; L-type lectin-domain containing receptor kinase
1	52.88–55.32	C2019; G2021	Serine/threonine-protein kinase; RGA1
2	36.6–42.4	C2019; G2021	Disease resistance proteins RGA1 and RPS2
3	14.53	C2022	Peroxidase
4	7.78–16.08	C2019; 2021;2022	RGA2; Zinc finger BED domain-containing protein; LRR-receptor-like protein kinase
4	47.26–49.48	C2021; G2022	Putative disease resistance RPP13-like protein; Pathogenesis like protein 5
5	70.24–76.94	C2019;2022	Zinc finger BED domain-containing protein; wall-associated protein kinases
6	0.05–2.19	C2021; G2021	Lipoxigenase
6	42.41	C2019	None
6	63.80-72.16	C2021; 2022	Peroxidase
7	12.63	G2021	Ras-related protein
7	59.35–86.92	C2019; 2021; G2021	RGA1; RGA3; RPP13-like protein; Serine/threonine-protein kinase; Protein kinase PP2C-WAT
8	27.16–39.65	C2019; 2021	F-box protein; Auxin response SAUR32
9	5.10–6.87	C2019; 2021	Receptor-like cytosolic serine/threonine-protein kinase
10	17.84	C2021	Probable RNA-binding protein ARP1
11	62.6–71.2	G2021	RGA3; Wall-associated receptor kinase
12	7.55	C2019	Serine/threonine-protein kinase; LRR-receptor-like protein kinase
12	35.56	C2019	None
14	3.6–6.18	C2021	LRR protein
14	55.19	C2021	PBS1 resistance gene; Zinc finger BED domain-containing protein
15	9.9–15.6	C2021; 2022; G2021	LRR-receptor-like serine/threonine-protein kinase; Zinc finger BED domain-containing protein; L-type lectin-domain containing receptor kinase
16	1.7–3.9	C2021; G2021	Serine/threonine-protein kinase
17	76.3-78.27	C2021	RGA2; Wall-associated receptor kinase
18	17.45	C2019	None

*Chr*, Chromosome. *G*, Guadajira. *C*, Córdoba

### Inheritance studies

In 2019, the parental line Q122 was susceptible to *P. variabilis*, showing at the end of the disease assessment period, on average, 28.3% of its area covered by the disease and sporulation. By contrast, plants of the resistant parent PI614911 were indeed highly resistant, showing no symptoms or, at maximum, a few scattered yellow spots caused by the disease and no sporulation. The F_2_ population Q122 x PI614911 segregated into 92 resistant and 30 susceptible, fitting perfectly the 3:1 ratio expected for a single dominant gene (χ2 = 0.01; *p* = 0.92) (Table 3). Differences between resistant and susceptible plants were evident as resistant plants showed as maximum a few scattered spots caused by the disease while susceptible plants were at least as susceptible as their susceptible parent. The phenotype of the F_2_ plants was confirmed evaluating their derived F_3_ families during 2020. All F_2_ resistant plants produced F_3_ families that were resistant or segregated for resistance, while all F_3_ families derived from F_2_ susceptible plants were susceptible. However, due to severe problems with the emergence of the seeds, for most families less than 10 F_3_ plants emerged. In order to obtain accurate conclusions, we only considered for the resistance segregation analysis those families having at least 10 plants. Of the 56 F_3_ families having at least 10 plants, 12 were resistant, 12 were susceptible and 32 segregated for resistance. These ratios also fit to the 1:2:1 ratio expected for a single dominant gene (χ2 = 1.14; *p* = 0.56). The ratio of resistant plants to susceptible plants in the 32 families that segregated for resistance was consistent with the hypothesis of the existence of a major dominant gene controlling resistance.

### Bulk segregant analysis (BSA)

**Fig. 3:**
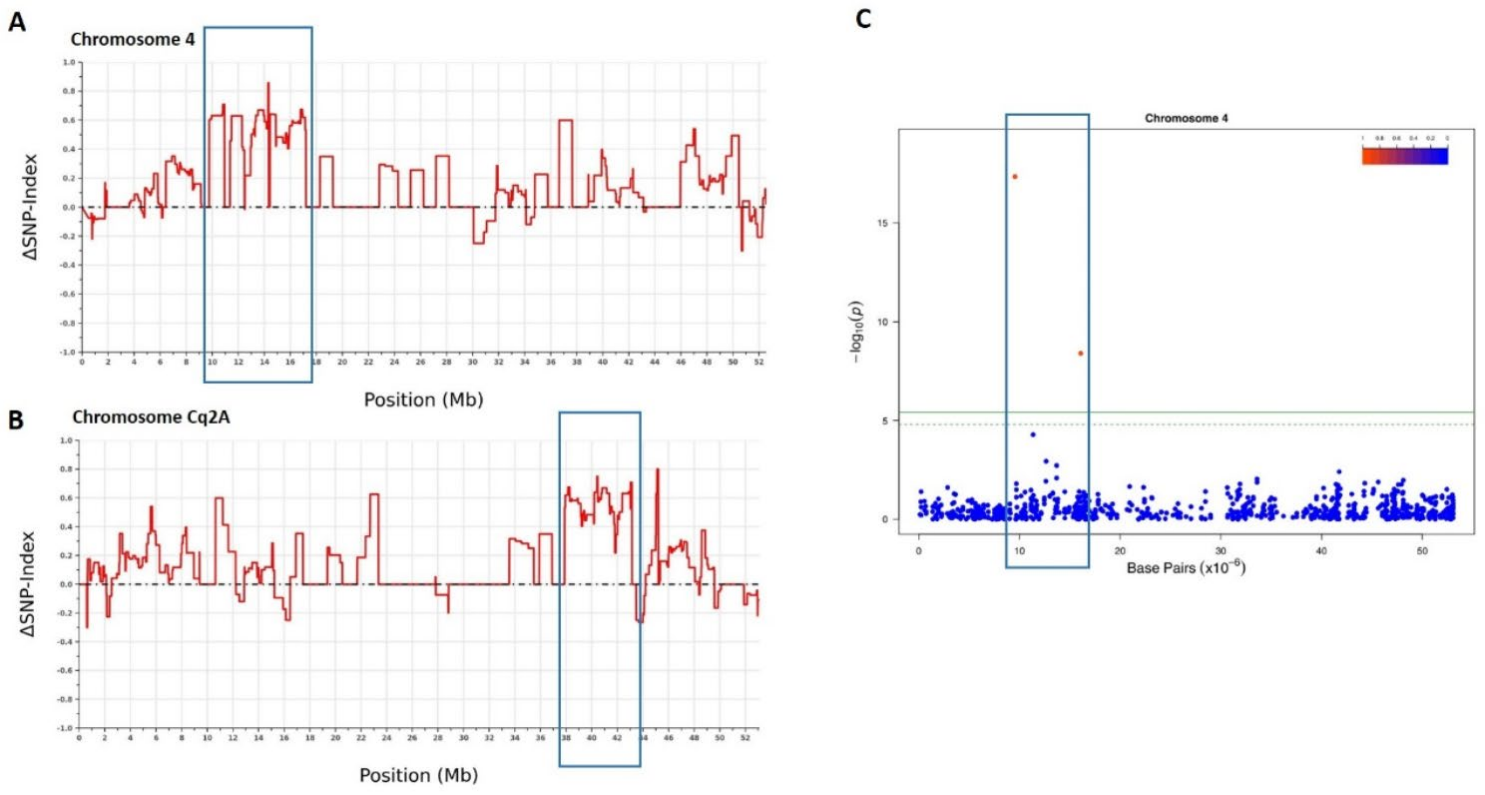
Genomic region containing the major gene conferring resistance to downy mildew in accession PI614911 identified by **(A)** BSA using version one of quinoa reference genome CoGe id33827 **(B)** BSA using version two of quinoa reference genome (CoGe id60716) **(C)** GWAS analysis performed using GAPIT software, SilicoDart markers and BLINK model. Manhattan plot for chromosome 4 (version one of quinoa reference genome) is shown. Chromosome 4 in version one of the quinoa reference genome corresponds to chromosome Cq2A in version two of the quinoa reference genome, and the region highlighted in **(A)** corresponds to the same region highlighted in **(B)**

The segregating F_2_ population derived from the cross between the susceptible line Q122 and the resistant PI614911, described above, was used to perform a BSA. After eliminating those SNPs that could not be assigned to chromosomes, a total of 11,418 SNPs were included in the analysis. ΔSNP-index graph across the different chromosomes identified a region showing an increase in ΔSNP-index values in the region 10.3–16.4 Mb on chromosome 4 (version 1 of quinoa reference genome CoGe id33827). In this region average ΔSNP index was high, being > 0.7 for 13 SNPs. This profile was not observed in any other region of the genome. Taking into account that in BSA, for a F_2_ population, the ΔSNP-index threshold to consider an imbalance of allele frequencies is 0.67 [36], these results demonstrate that the dominant gene controlling resistance to downy mildew in accession PI614911 is located into this region. SNPs identified by DArTseq sequencing were further positioned on the version two of quinoa reference genome (CoGe id607169) and a ΔSNP-index graph was also created. In agreement with the previous results, region 38.64–42.51 Mb on chromosome Cq2A, that corresponds to the region identified as a carrier of the resistance gene in version one of quinoa reference genome, also showed a clear increase in ΔSNP index (Fig. 3). Furthermore, this region is included into a region identified in the GWAS analysis performed in our study, confirming both, GWAS and BSA studies.

**Table 3:** Segregation of resistance to *P. variabilis* in Q122* PI614911 cross

Cross	Generation	No plants/families evaluated	Observed ratio	Expected ratio	χ^2^	Probability
Q122* PI614911	F_2_	122	92:30	91.5:30.5	0.01	0.92
	F_3_	56	12:32:12	14:28:14	1.14	0.56

## Discussion

Despite the relevance of downy mildew disease in quinoa little is known about the genetics of downy mildew resistance in quinoa. Knowledge of the genetic control of this trait would be useful to plan the best strategy to incorporate this trait into elite cultivars. The only study analysing the inheritance of *P. variabilis* resistance was performed by [17]. The authors evaluated the response to this disease in a recombinant inbred line population derived from a cross between a slightly susceptible accession and a resistant accession. In this population the trait behaved as a quantitative trait and transgressive segregation was observed, suggesting that resistance was polygenic and that the parents harboured different resistant genes. By contrast, in our study we have identified complete qualitative downy mildew resistance and demonstrated that this resistance is controlled by a single dominant gene in quinoa accession PI614911. Single gene-controlled resistance is easy to incorporate into susceptible material through backcrossing. Therefore, the identification of a major gene conferring complete downy mildew resistance in quinoa is a milestone that will greatly facilitate the development of resistant cultivars. So that, we have already used accession PI614911 to successfully incorporate resistance to downy mildew in some of our more interesting advanced breeding material.

Furthermore, through a BSA analysis, we have identified the genomic region containing this major gene. The resolution of BSA is expected to be lower than GWAS because the number of generations over which the population is interbred is limited [37]. However, BSA was useful to corroborate GWAS results and discern which of the different genomic regions identified by GWAS was responsible for resistance in accession PI614911. Our results reveal that the gene conferring resistance in accession PI614911 is located in the region 10.3–16.4 Mb on chromosome 4 (version one of quinoa reference genome). This region corresponds to the region 38.9–42.8 Mb on Chromosome Cq2A in version 2 of the quinoa reference genome (CoGe id60716) (Fig. 3), a region that was also postulated to be associated with resistance to downy mildew in the GWAS analysis performed by [19]. These results suggest that this resistance gene is not a rare gene but, rather, a gene that may be present in several quinoa accessions. Exciting, a gene predicted to be similar to “Disease resistance protein RGA2” is located exactly in the same positions as the MTA identified at 16,082,246 pb on Chromosome 4. This gene is an excellent candidate for this major resistance gene. Further sequencing of this candidate gene and gene expression studies, in PI614911and susceptible lines, and mapping in segregating populations are planned to confirm this hypothesis. Other interesting candidate genes located in this region are different types of “Receptor-like protein kinases” and a “Zinc finger BED domain-containing protein” (Suppl. Table S4).

The identification of both, quantitative and qualitative resistance to downy mildew in our quinoa collection suggest that both, major and minor genes may be involved in resistance to this pathogen depending on the accession. In agreement, in addition to the region on chromosome 4 commented above, our GWAS analysis identified several other regions associated with downy mildew resistance. Therefore, additionally to the major gene present in accession PI614911, there are, probably, other genes conferring resistance to this important disease in quinoa germplasm. In a previous study [16], we demonstrated the presence and high relevance of hypersensitive response as a defense mechanism against *P. variabilis* in quinoa. Hypersensitive response, which is a pathogen-induced cell death process at the site of infection that limits pathogen growth, is a common mechanism of resistance against biothrophic pathogens, as downy mildew. HR is the result of the recognition of pathogen effectors by the plant, unleashing effector-triggered immunity (ETI), and is activated by R-genes. In agreement with that, many of the regions associated with resistance to *P. variabilis*, according to our GWAS analysis, harbour plant receptor genes or resistance genes (Table 2). Especially exciting is the presence of nine of these genes located exactly in the same position as the MTAs identified by GWAS. These genes include two genes annotated as “disease resistance RPP13-like protein”, the gene annotated as RGA2 mentioned above as candidate gene for the major gene conferring resistance to downy mildew in accession PI614911, one gene annotated as RGA1, other gene annotated as RGA3, another annotated as “serine/threonine-protein kinase”, a “F-box/LRR-repeat protein”, a “LRR receptor-like serine/threonine-protein kinase” and a “wall-associated-receptor kinase” encoding genes (Suppl. Table S4). Plant resistance gene analogs (RGAs) act as intracellular receptors that perceive the presence of pathogen effectors by direct binding of the pathogen effector proteins, or by monitoring the modification of host proteins after associating with the pathogen, to activate multiple defense signal transductions to restrict pathogen growth [38]. RGAs include nucleotide binding site leucine rich repeats, receptor like kinases, receptor like proteins, pentatricopeptide repeats and apoplastic peroxidases [39]. The presence of genes similar to RPP13 in two MTAs is especially attractive, since RPP13 is a resistance gene that confers resistance to downy mildew in *Arabidopsis* [40]. Downy mildew in Arabidopsis is caused by *Peronospora parasitica*, a member of the same genus as the pathogen causing downy mildew in quinoa (*Peronospora variabilis*).

Another remarkable outcome is the presence of genes encoding “Zinc finger BED domain-containing protein” in the genomic regions associated with resistance identified by GWAS. BED domains have been found to be integrated into plant resistance genes from different plant species [41]. This kind of genes are frequent in quinoa genome, however, the presence of this kind of genes in more than one candidate region associated with resistance suggest that this type of genes can also play a relevant role in resistance against downy mildew in quinoa.

To validate the candidate genes identified in our GWAS analysis, the same approach as mentioned for the major gene conferring downy mildew resistance in accession PI614911 could be followed. That is, sequencing these genes and performing gene expression studies in accessions showing contrasting profiles for the MTAs associated.

Disease severity values were correlated between scorings performed in the same location. A Pearson’s correlation coefficient as high as 0.85 was found between scorings carried out in Córdoba in 2019 and 2022, demonstrating the accuracy of the method used to evaluate the response to the disease. However, disease severity between different locations was not correlated. Furthermore, the analysis of variance performed also indicated that disease severity was influenced by the location (Suppl. Table S2). In agreement with that, according to GWAS, some genomic regions were found to be associated with resistance to downy mildew only in one location. This differential response to downy depending on the location suggest the presence of *P. variabilis* races/isolates with different virulence in Córdoba than in Guadajira. The presence of races in the pathosysthem *P. variabilis-Chenopodium quinoa* was already suggested by Ochoa et al. (1999). Reinforcing this hypothesis, in a previous article [16], we already commented different reactions to *P. variabilis* (complete resistance vs. high susceptibility) of some quinoa accessions in the screenings performed in Córdoba compared with screenings carried out in other countries. The presence of R-genes into the genomic regions associated with resistance (that are typically race-specific) and the existence of HR in *P. variabilis-C. quinoa* (a mechanism present frequently in race-specific resistance) reinforce this hypothesis. Therefore, the genomic regions associated with resistance to downy mildew only in one location may harbour genes providing race-specific resistance to the *P. variabilis* races present in one region but, that can be overcome by other races with different virulent pattern present in other regions. PCA and fastSTRUCTURE divided the quinoa collection in two and four populations, respectively. Interestingly, there was a correlation between the response of the quinoa accessions to *P. variabilis* in each location and the belonging population (Suppl. Table S5), supporting the hypothesis of a differential pattern of resistance genes depending on the genetic population. So that, in general, quinoa accessions belonging to population 2 according to PCA (corresponding to highland quinoas), were more susceptible to downy mildew than those belonging to population 1 (corresponding to lowland quinoas) in Córdoba (Suppl. Table S5). Thus, the difference in average disease severity between lowland and highland accessions was statistically significant for the years 2021 and 2022. The opposite trend was observed in the case of Guadajira, where lowland quinoas were, as average, more susceptible than highland ones. In 2019, the same trend was observed, although differences were not statistically significant. In agreement, regarding the four populations identified by fastSTRUCTRE, lowland accessions, belonging to population 4, were, on average, the most susceptible accessions in Guadajira. Highland accessions were divided in 3 subpopulations in the analysis performed by fastSTRUCTRE and, similarly, population 2, corresponding to highland accessions mainly from Bolivia, was the population that showed higher disease severity, on average, in Córdoba (Suppl. Table S5).

## Conclusion

Despite the relevance of downy mildew disease in quinoa, little was known about the genetic control of resistance to this disease. We here identified a set of genomic regions associated with this trait and provide plausible candidate genes located within these regions. The enrichment of these regions in plant receptors and resistance genes point out to a high relevance of gene-by-gene interactions controlling resistance/susceptibility to *P. variabilis* in quinoa. What more, one of these regions identified by GWAS was confirmed by BSA, and found to harbour a single dominant gene conferring complete resistance to downy mildew. All these outcomes markedly increased our current knowledge about the genetics of resistance to downy mildew in quinoa, providing valuable information for breeding for resistance to this important disease.

### Electronic supplementary material

Below is the link to the electronic supplementary material.

Supplementary Material 1

Supplementary Material 2

Supplementary Material 3

Supplementary Material 4

Supplementary Material 5

Supplementary Material 6

Supplementary Material 7

Supplementary Material 8

Supplementary Material 9

## Data Availability

The raw sequencing data that support the findings of this study have been deposited in the NCBI Sequence Read Archive (SRA) under the BioProject PRJNA1040439.
