# Supplementary figures and images for: Genome-wide association study, combined with bulk segregant analysis, identify plant receptors and defense related genes as candidate genes for downy mildew resistance in quinoa
Source: BMC Plant Biol. 2024 Jun 24;24:594. doi: 10.1186/s12870-024-05302-2 (PMC11194881; doi:10.1186/s12870-024-05302-2)

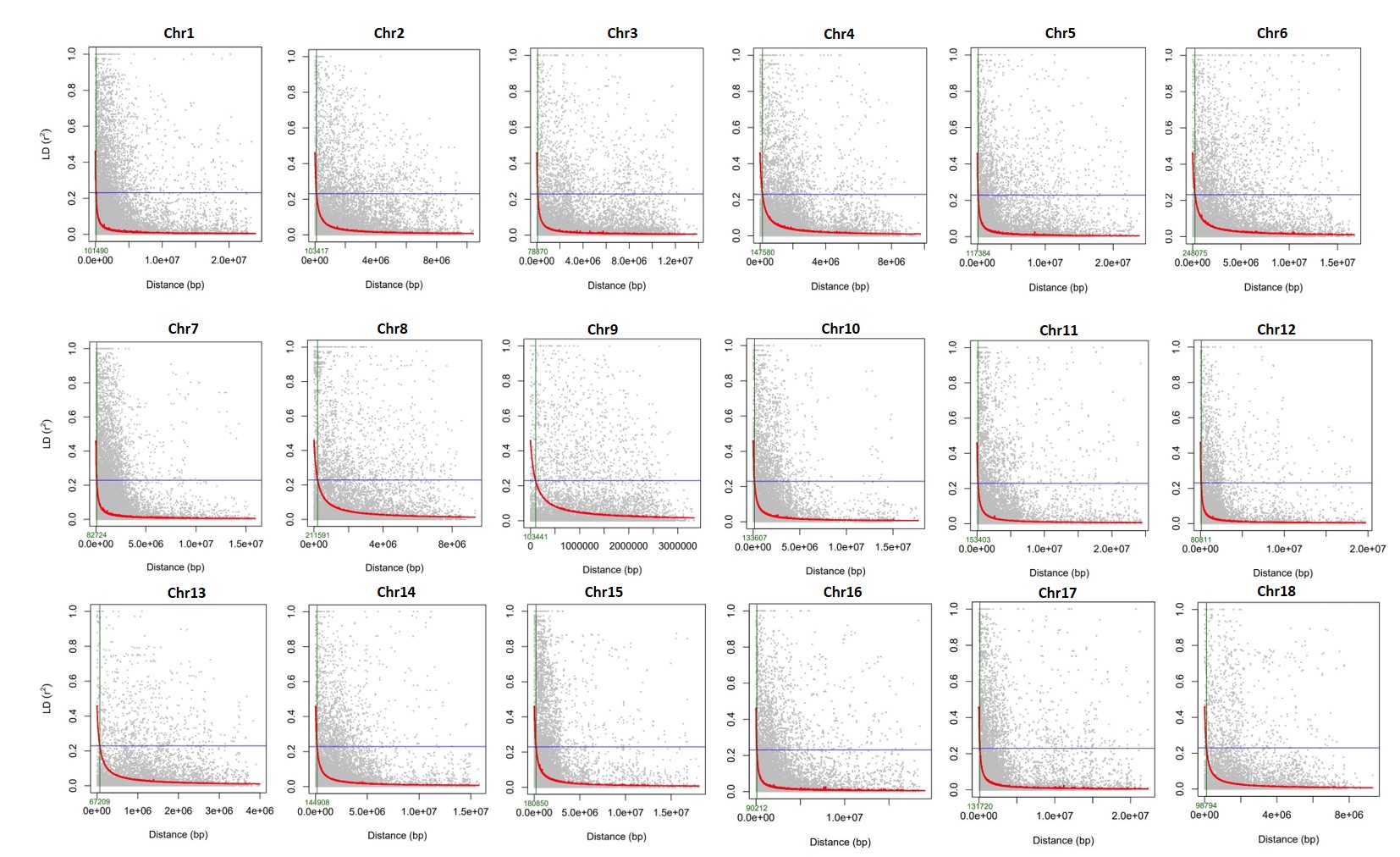

Supplement: Supplementary file 1 — Supplementary Material 1 [file 12870_2024_5302_MOESM1_ESM.jpg]

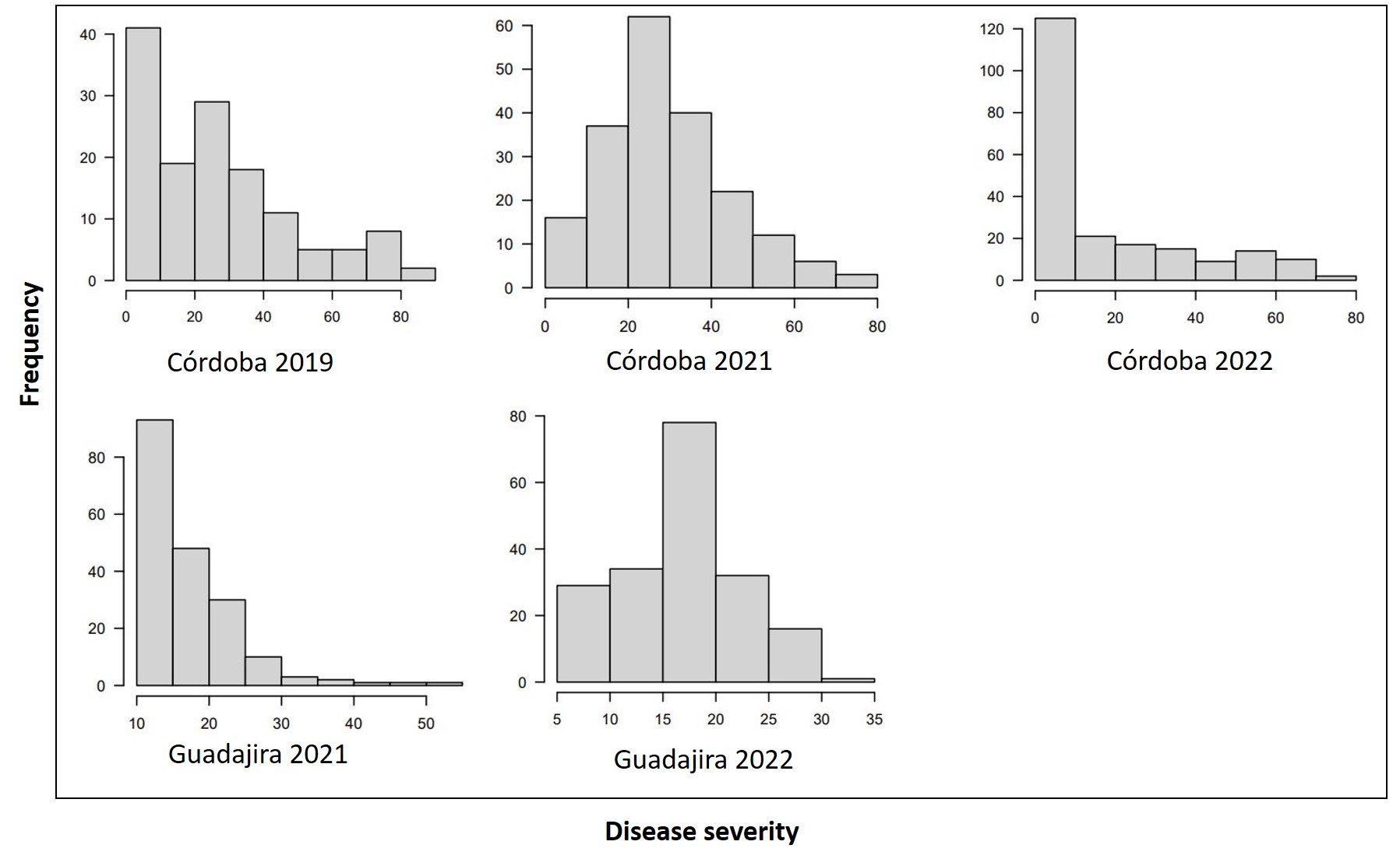

Supplement: Supplementary file 2 — Supplementary Material 2 [file 12870_2024_5302_MOESM2_ESM.jpg]

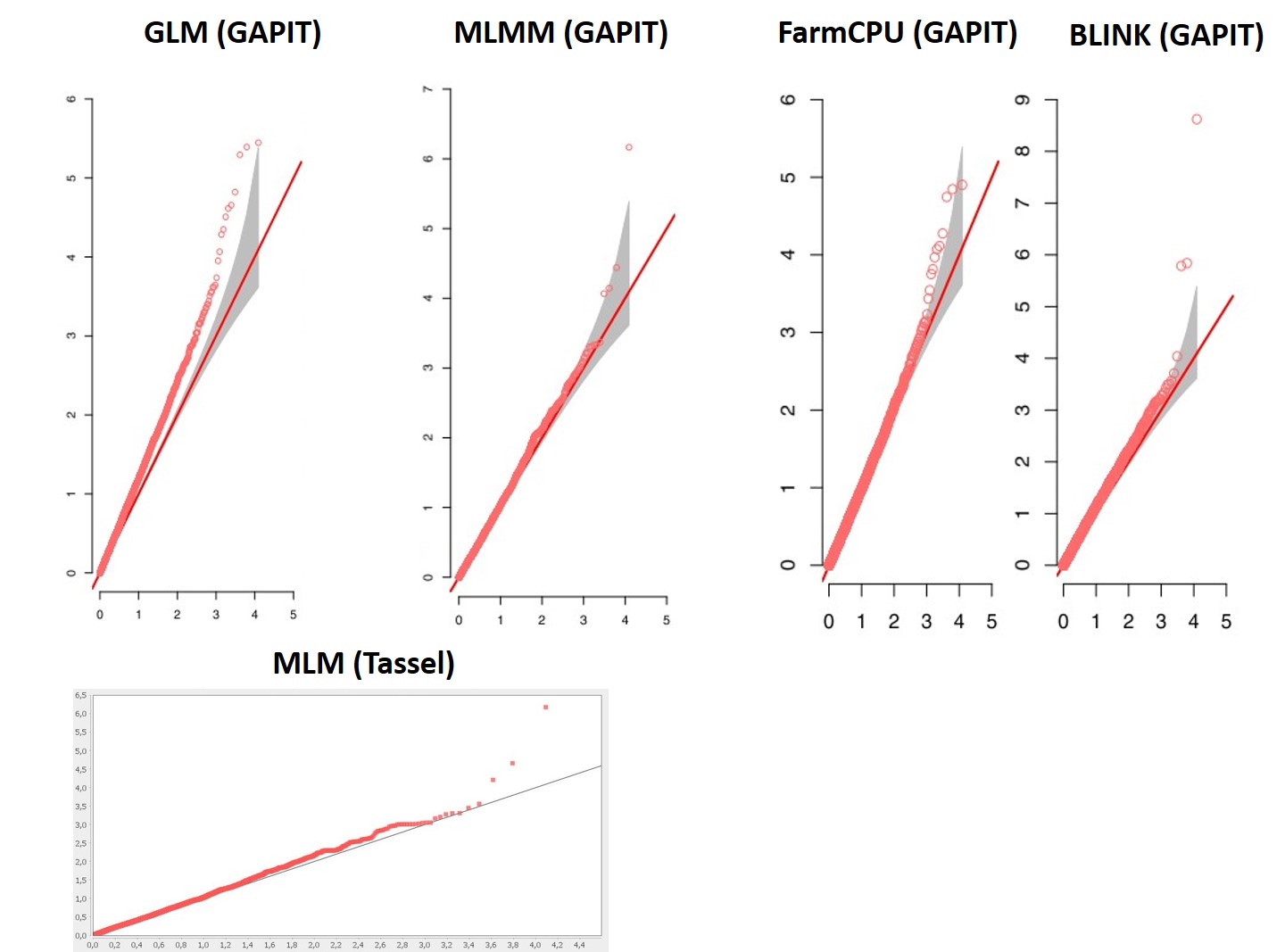

Supplement: Supplementary file 3 — Supplementary Material 3 [file 12870_2024_5302_MOESM3_ESM.jpg]
