# Supplementary material for: Genome-wide association study, combined with bulk segregant analysis, identify plant receptors and defense related genes as candidate genes for downy mildew resistance in quinoa
Source: BMC Plant Biol. 2024 Jun 24;24:594. doi: 10.1186/s12870-024-05302-2 (PMC11194881; doi:10.1186/s12870-024-05302-2)
Supplement: Supplementary file 5 — Supplementary Material 5 [file 12870_2024_5302_MOESM5_ESM.docx]

**Supplementary Table S2** Analysis of variance for the variable ‘downy mildew final disease severity’ scored in a collection of quinoa accessions in two locations during three years

| **Source** | **SS** | **gl** | **MS** | **F** | **p-value** |
| --- | --- | --- | --- | --- | --- |
| Accession | 102471.991 | 104 | 985.308 | 18.913 | 0.000 |
| Location | 380.795 | 1 | 380.795 | 7.309 | 0.007 |
| Year | 18780.669 | 2 | 9390.334 | 180.247 | 0.000 |
| Accession * Location | 62258.308 | 104 | 598.638 | 11.491 | 0.000 |
| Accession *Year | 60556.138 | 208 | 291.135 | 5.588 | 0.000 |
| Location * Year | 9155.656 | 1 | 9155.656 | 175.742 | 0.000 |
| Accession * Location * Year | 26733.512 | 104 | 257.053 | 4.934 | 0.000 |
| Error | 48658.667 |  | 52.067 |  |  |
| Total | 1052977.000 |  |  |  |  |
