# Supplementary material for: Genome-wide association study, combined with bulk segregant analysis, identify plant receptors and defense related genes as candidate genes for downy mildew resistance in quinoa
Source: BMC Plant Biol. 2024 Jun 24;24:594. doi: 10.1186/s12870-024-05302-2 (PMC11194881; doi:10.1186/s12870-024-05302-2)
Supplement: Supplementary file 6 — Supplementary Material 6 [file 12870_2024_5302_MOESM6_ESM.docx]

**Supplementary Table S3** Number of SNPs and SilicoDArT markers used in the GWAS analysis, and the average distance between markers, per chromosome.

| **Chromosome** | **SNP** | **SilicoDArT** | **Total** | **Average distance (Kb)** |
| --- | --- | --- | --- | --- |
| 1 | 1292 | 1342 | 2634 | 50.02 |
| 2 | 719 | 674 | 1393 | 42.44 |
| 3 | 621 | 733 | 1354 | 59.15 |
| 4 | 758 | 888 | 1646 | 32.16 |
| 5 | 647 | 674 | 1321 | 62.09 |
| 6 | 541 | 621 | 1162 | 63.93 |
| 7 | 1449 | 1467 | 2916 | 38.5 |
| 8 | 529 | 540 | 1069 | 41.62 |
| 9 | 317 | 240 | 557 | 26.09 |
| 10 | 715 | 604 | 1319 | 45.73 |
| 11 | 614 | 675 | 1289 | 57.82 |
| 12 | 657 | 695 | 1352 | 42.5 |
| 13 | 284 | 260 | 544 | 26.69 |
| 14 | 595 | 582 | 1177 | 50.22 |
| 15 | 811 | 995 | 1806 | 34.35 |
| 16 | 742 | 639 | 1381 | 57.25 |
| 17 | 698 | 710 | 1408 | 59.32 |
| 18 | 408 | 381 | 789 | 41.35 |
