# Supplementary material for: Genome-wide association study, combined with bulk segregant analysis, identify plant receptors and defense related genes as candidate genes for downy mildew resistance in quinoa
Source: BMC Plant Biol. 2024 Jun 24;24:594. doi: 10.1186/s12870-024-05302-2 (PMC11194881; doi:10.1186/s12870-024-05302-2)
Supplement: Supplementary file 9 — Supplementary Material 9 [file 12870_2024_5302_MOESM9_ESM.docx]

**Supplementary Materials**

**Supplementary Table S1** Code, origin and population group according to PCA and fastSTRUCTURE, of quinoa accessions included in the study.

**Supplementary Figure S1** Linkage Desequilibrium Decay in the different quinoa chromosomes (Chr). LD half decay distance is shown in green.

**Supplementary Table S2** Analysis of variance for the variable ‘downy mildew final disease severity’ scored in a collection of quinoa accessions in two locations during three years

**Supplementary Figure S2** Histograms indicating the number of accessions (frequency) displaying the different levels of ‘downy mildew disease severity’ in a collection of quinoa accessions in Córdoba (years 2019, 2021 and 2022) and Guadajiara (years 2021 and 2022).

**Supplementary Table S3** Number of SNPs and SilicoDArT markers used in the GWAS analysis, and the average distance between markers, per chromosome.

**Supplementary Figure S3** Quantile-quantile plot (QQplot) comparing observed - log10 (P) for each marker (X axis) against expected - log10 (P) values under the null hypothesis (no-association of the marker with the trait) (Y axis). QQplots shown were obtained from GWAS analysis using GAPIT and SNPs markers, for the trait “Disease severity Córdoba 2022”, using the models GLM, MLM, MLMM, FarmCPU and BLINK.

**Supplementary Table S4** MTAs identified by GWAS.

**Supplementary Table S5** Analysis of the relationship between the response to *P. variabilis* and the population of belonging.
